# Detection and molecular characterization of *Trichomonas gallinae* recovered from domestic pigeons in Egypt

**DOI:** 10.1007/s00436-022-07724-z

**Published:** 2022-11-25

**Authors:** Hend M. Mohamed, Aalaa S. A. Saad, Marwa M. Khalifa, Sahar Z. Abdel-Maogood, Salwa M. F. Awadalla, Waheed M. Mousa

**Affiliations:** 1 Poultry Diseases Department, Animal Health Research Institute (AHRI), Agriculture Research Center (ARC), Dokki, Egypt; 2 Biotechnology Department, Animal Health Research Institute (AHRI), Agriculture Research Center (ARC), Dokki, Egypt; 3 Parasitology Department, Faculty of Veterinary Medicine, Cairo University, Giza, Egypt

**Keywords:** *Trichomonas gallinae*, Canker, Modified Diamond’s media, PCR

## Abstract

*Trichomonas gallinae* is a protozoan parasite that causes canker in pigeons. Squabs (young pigeons) are frequently infected with *T. gallinae* and can die because of the infection, while adult pigeons can act as carriers showing no clinical signs. In the present study, 50 squabs, up to 1-month-old, were purchased from pigeon markets in different regions of the Giza governorate, Egypt. Direct wet mount preparations of the oral excretions of the squabs (mouth wash) and Giemsa staining revealed that 64% (32/50) were positive for *T. gallinae*. Experimental infection of ten squabs with 10^3^ *T. gallinae* trophozoites/ml resulted in oral lesions on the mouth, tongue, and soft palate, with the presence of yellowish-white nodules (cheese-like) in the oral cavity on the sixth day post-infection in all squabs. A subset of five samples were cultured in modified Diamond’s media, their DNA was extracted, and a portion of the ribosomal internal transcribed spacer region (ITS1/5.8S/ITS2) was amplified by polymerase chain reaction (PCR) followed by sequencing. Phylogenetic analysis of the five isolates revealed 64–91% homology with some reference isolates circulating in Egypt and related countries.

## Introduction

Avian trichomoniasis is a parasitic protozoan disease that affects pigeons, doves, chickens, turkeys, and raptors (Bulbul et al. 2018). The disease is called canker in pigeons (Saikia et al. 2021) and frounce in birds of prey. The causative agent, *Trichomonas gallinae*, is a flagellate belonging to the family Trichomonadidae, order Trichomonadida. Recently, two new species were recognized: *T. stableri* (Girard et al. 2014) and *T. gypaetinii* (Martínez-Díaz et al. 2015).

*T. gallinae* inhabits the upper digestive tract, mostly the esophagus and crop, but it can also infect the lungs, liver, internal lining of the body, air sacs, pancreas, bones, and skull sinuses. The disease is transmitted to birds through various routes, including crop milk, billing or feeding courtship rituals, aggregation at bird feeders or contaminated birdbaths, and the consumption of infected prey (Grunenwald et al. 2018).

The disease can be diagnosed in the laboratory by molecular identification of the organism and by clinical signs. Due to its low sensitivity, the wet mount method cannot distinguish strains of *Trichomonas* spp. Using molecular data allows the assessment of phylogenetic relationships among similar organisms (Purple 2018).

Recently, molecular techniques have been employed to characterize this parasite and establish relationships between isolates (Hochleithner and Hochleithner 2021). Low amounts of *Trichomonas* spp. can be detected due to the sensitivity of the polymerase chain reaction (PCR) to identify parasite DNA. Various DNA targets, including the internal transcribed spacer region (ITS), 18S rRNA, and iron hydrogenase, have proven effective for identifying trichomonads and for differentiating strains (McBurney et al. 2015). The present study aimed to investigate the isolated *T. gallinae* parasite by PCR, perform sequencing analyses targeting the ITS region, and compare the Egyptian *T. gallinae* sequences with those from other countries.

## Materials and methods

### Collection of pigeon samples and study region

Fifty squabs, up to 1-month-old, showing signs of depression, weakness, anorexia, ruffled feathers, reluctance to fly, and caseated material in the oral cavity were purchased from Giza governorate, Egypt pigeon markets, from September–December 2021.

### Microscopic examination for detection of *T. gallinae* trophozoites and staining method

In the direct wet mount method, Florin-Christensen and Schnittger (2018) sampled oral excretions (mouth wash) from squabs and checked for motile trophozoites within 30 minutes under a light microscope at 10x and 40x magnifications. They were identified by their motility and some morphological features, such as being pyriform to round, 7–11 μm in size, having four free flagellae, a well-developed undulating membrane, and an oval nucleus.

Then, the slides were Giemsa-stained and examined using an oil immersion lens (100x) according to Hamad and Hassan (2017).

### Preparation of *T. gallinae* culture and experimental infection

Ten positive samples of *T. gallinae* were selected, and individually cultivated in modified Diamond’s media (MD, trypticase yeast extract media) prepared according to Raza et al. (2018).

Motile trophozoites were counted using a hemocytometer (Neubauer Improved, Germany) at 40x magnification, according to Hamad and Hassan (2017), by the following equation:

$$\mathrm{No}.\;\mathrm{of}\;\mathrm{motile}\;\mathrm{trophozoites}/\mathrm{ml}=\mathrm{no}.\;\mathrm{of}\;\mathrm{counted}\;\mathrm{trophozoites}\times10^4$$

The media was inoculated with 2 × 10^5^ trophozoites/ml, the inoculated tubes were tightly capped, incubated at 37 °C, and examined daily for 5 days. Only motile *T. gallinae* were estimated.

Each of the five positive samples was selected at random from the ten samples of *T. gallinae* cultivated in MD media, and was inoculated individually into ten healthy squabs for up to 1 month. The squabs had been collected from Giza governorate markets and subjected to parasitological examination to confirm they were infection-free.

They had been reared under entirely hygienic conditions and were infected orally with 1 ml of 10^3^ T*. gallinae* trophozoites/ml using a dropper (Mohamed et al. 2009). Post-infection, the samples were collected from the oral cavity daily and examined via a direct wet smear.

### Sampling *T. gallinae* for PCR and DNA extraction

*T. gallinae* trophozoites were collected from experimentally infected squabs, counted and adjusted to 2 × 10^5^ cells/ml, and cultured in MD media at 37 °C for 48 h until the count of motile trophozoites equaled 1.49 × 10^6^.

The cultures were centrifuged at 1500 × g for 10 min (Echenique et al. 2020) to obtain the five isolates, the supernatants were discarded, and the pellets were re-suspended in phosphate-buffered saline, and stored at − 20 °C until their DNA was extracted.

### PCR amplification of the ITS1/5.8S/ITS2 fragment

Genomic DNA was extracted from the five isolates using the EasyPure® Genomic DNA Kit (China), according to the manufacturer’s instructions. The concentration of the extracted DNA was measured on the Nanodrop 2000 micro-volume UV–VIS spectrophotometer (Thermo Scientific, USA).

Quantitative PCR was performed using HERA SYBR® Green RT-qPCR, the forward primer TFR1 (5′-TGCTTCAGTTCAGCGGGTCTTCC-3′), and the reverse primer TFR2 (5′-CGGTAGGTGAACCTGCCGTTGG-3′), to amplify the target DNA sequence (El-Khatam et al. 2016; Albeshr and Alrefaei 2020).

### Sequencing the ITS1/5.8S/ITS2 amplicons

Positive PCR products were sent to Macrogen® Company for double-strand sequencing using an ABI 3730xl DNA Sequencer. The sequencing data were analyzed using NCBI Blast (Altschul et al. 1990), assembled, edited, and chromate graphed using the Jalview software version 1.8.3–1.2.9-JAL.

The phylogenetic tree was created using the MegAlign module. Neighbor-joining phylogenetic analyses were performed in MEGA X: Molecular Evolutionary Genetics Analysis across computing platforms (Kumar et al. 2018). The tree was rooted in the outgroup, *Tetratrichomonas* spp.

## Results and discussion

Of 50 squabs, 32 tested positive for *T. gallinae*, as assessed by direct wet mount preparations. *T. gallinae* possesses four unequal, anterior, transparent flagella that may be single or in groups, a well-developed fin-like undulating membrane, and an oval nucleus. Giemsa-stained the nucleus and flagella light purple while the cytoplasm was stained dark purple. In the present study, the microscopic structures of *T. gallinae* resembled those described by Heinz (2016).

Cultivation of *T. gallinae* in MD media, with an initial inoculum of 2 × 10^5^ cells/ml, produced the maximum growth with hyperactivity in movement at 48 hours post-incubation, when the trophozoite count reached 1.49 × 10^6^ trophozoites/ml. This agrees nearly with the estimation of 1.325 × 10^6^ cells/ml reported by Hamad and Hassan (2017). Diamond’s medium has been the most widely used diagnostic standard culture medium for the identification and propagation of *T. gallinae*. Fresh inactivated horse serum, a rich source of amino acids, fatty acids, and some trace elements, is an essential additive for the growth of *Trichomonas* species in culture (Raza et al. 2018).

**Fig. 1:**
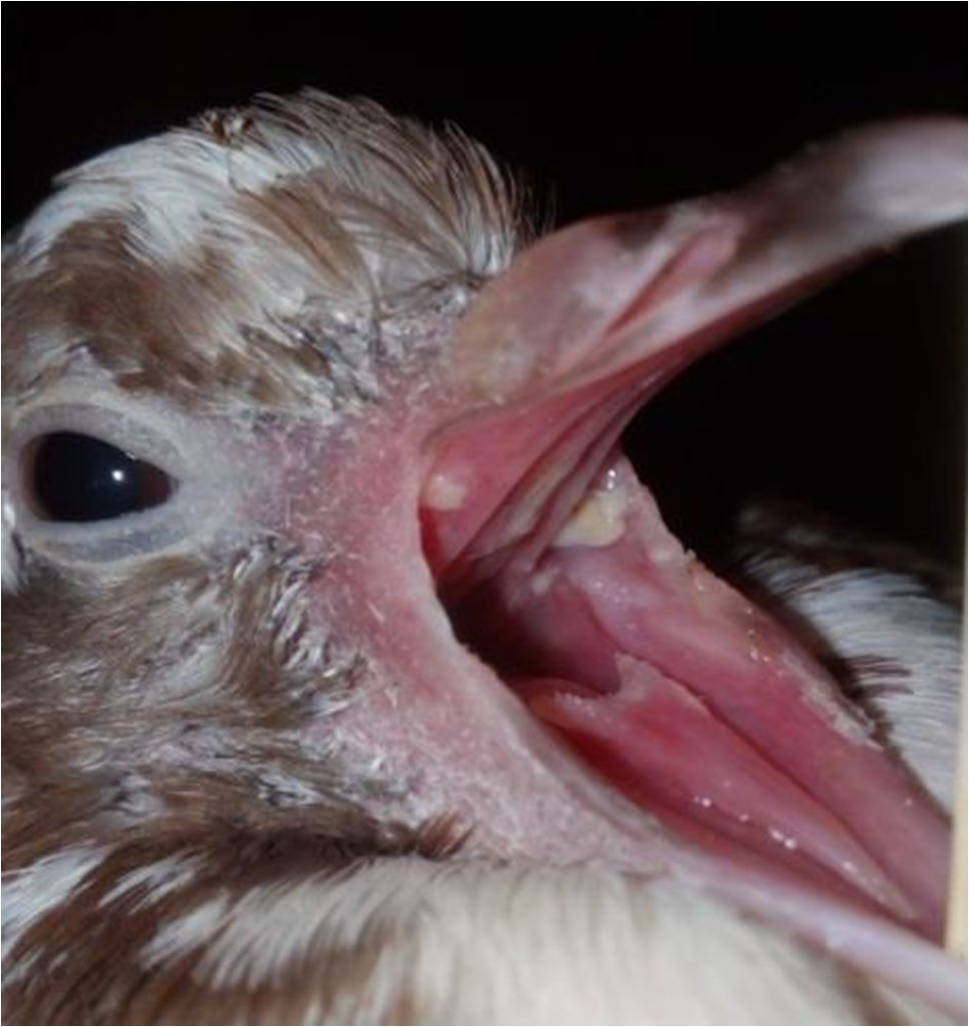
Clinical gross lesions examination of squab on the sixth day post-infection showed the presence of yellowish-white caseated nodules in the oral cavity

Experimentally infected squabs showed oral lesions on the mouth, tongue, and soft palate, and yellowish-white nodules (cheese-like) in the oral cavity on the sixth day post-infection (Fig. 1). The oral lesions began as small, white caseous nodules, which subsequently grew into large yellowish-white caseous nodules. Similar observations were reported by Fadhil et al. (2020).

**Fig. 2:**
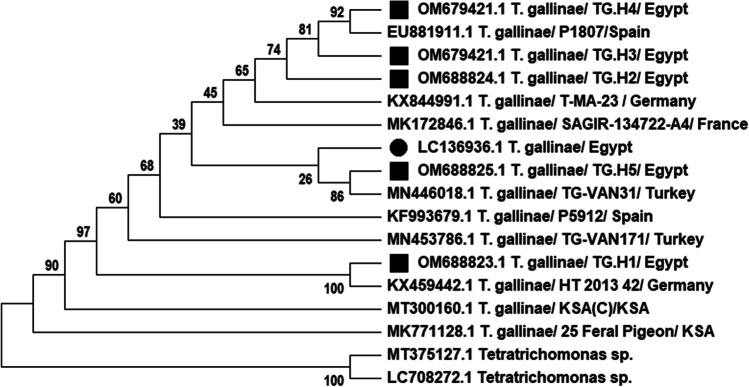
Phylogenetic relationships among *T. gallinae* strains OM688823 (TG.H1), OM688824, OM679421, OM679422, and OM688825 based on the alignment of the amplified ITS1/5.8S/ITS2 sequences. The tree is rooted by the outgroup (*Tetratrichomonas* spp.). The tree was constructed by the neighbor-joining method in the MEGA X. ▀ This study isolates; ● Egyptian isolate

The ITS1/5.8S/ITS2 sequences of the five examined *T. gallinae* isolates were submitted to GenBank under accession numbers OM688823, OM688824, OM679421, OM679422, and OM688825. The constructed phylogenetic tree (Fig. 2), as well as nucleotide identity analysis (Table 1), revealed different extents of homology between these isolates and other reference isolates circulating in Egypt and related countries.

**Table 1:** Nucleotide identities of this study isolates OM688823 (TG.H1), OM688824 (TG.H2), OM679421 (TG.H3), OM679422 (TG.H4), and OM688825 (TG.H5) with selected references and Egyptian strain sequence

Seq- >	OM688823.1 T. gallinae/TG.H1/Egypt	OM688824.1 T. gallinae/TG.H2/Egypt	OM679421.1 T. gallinae/TG.H3/Egypt	OM679422.1 T. gallinae/TG.H4/Egypt	OM688825.1 T. gallinae/TG.H5/Egypt	LC136936.1 T. gallinae/Egypt	MT300160.1 T. gallinae/KSA(C)/KSA	MK771128.1 T. gallinae/25_Feral_Pigeon/KSA
OM688823.1 T. gallinae/TG.H1/Egypt	ID	95%	70%	70%	94%	88%	61%	64%
OM688824.1 T. gallinae/TG.H2/Egypt	95%	ID	73%	73%	96%	90%	62%	63%
OM679421.1 T. gallinae/TG.H3/Egypt	70%	73%	ID	100%	69%	64%	85%	74%
OM679422.1 T. gallinae/TG.H4/Egypt	70%	73%	100%	ID	69%	64%	85%	74%
OM688825.1 T. gallinae/TG.H5/Egypt	94%	96%	69%	69%	ID	91%	63%	64%
LC136936.1 T. gallinae/Egypt	88%	90%	64%	64%	91%	ID	67%	70%
MT300160.1 T. gallinae/KSA(C)/KSA	61%	62%	85%	85%	63%	67%	ID	79%
MK771128.1 T. gallinae/25_Feral_Pigeon/KSA	64%	63%	74%	74%	64%	70%	79%	ID
KX459442.1 T. gallinae/HT_2013_42/Germany	100%	95%	70%	70%	94%	88%	61%	64%
MN453786.1 T. gallinae/TG-VAN171/Turkey	84%	85%	68%	68%	86%	94%	72%	74%
KF993679.1 T. gallinae/P5912/Spain	85%	87%	70%	70%	87%	91%	72%	73%
KX844991.1 T. gallinae/T-MA-23/Germany	92%	96%	69%	69%	96%	93%	64%	66%
MK172846.1 T. gallinae/SAGIR-134722-A4/France	94%	97%	70%	70%	98%	90%	61%	63%
EU881911.1 T. gallinae/P1807/Spain	94%	97%	72%	72%	95%	88%	61%	63%
MN446018.1 T. gallinae/TG-VAN31/Turkey	95%	96%	70%	70%	99%	91%	63%	64%

Seq- >	KX459442.1 T. gallinae/HT_2013_42/Germany	MN453786.1 T. gallinae/TG-VAN171/Turkey	KF993679.1 T. gallinae/P5912/Spain	KX844991.1 T. gallinae/T-MA-23/Germany	MK172846.1 T. gallinae/SAGIR-134722-A4/France	EU881911.1 T. gallinae/P1807/Spain	MN446018.1 T. gallinae/TG-VAN31/Turkey
OM688823.1 T. gallinae/TG.H1/Egypt	100%	84%	85%	92%	94%	94%	95%
OM688824.1 T. gallinae/TG.H2/Egypt	95%	85%	87%	96%	97%	97%	96%
OM679421.1 T. gallinae/TG.H3/Egypt	70%	68%	70%	69%	70%	72%	70%
OM679422.1 T. gallinae/TG.H4/Egypt	70%	68%	70%	69%	70%	72%	70%
OM688825.1 T. gallinae/TG.H5/Egypt	94%	86%	87%	96%	98%	95%	99%
LC136936.1 T. gallinae/Egypt	88%	94%	91%	93%	90%	88%	91%
MT300160.1 T. gallinae/KSA(C)/KSA	61%	72%	72%	64%	61%	61%	63%
MK771128.1 T. gallinae/25_Feral_Pigeon/KSA	64%	74%	73%	66%	63%	63%	64%
KX459442.1 T. gallinae/HT_2013_42/Germany	ID	84%	85%	92%	94%	94%	95%
MN453786.1 T. gallinae/TG-VAN171/Turkey	84%	ID	97%	88%	84%	84%	86%
KF993679.1 T. gallinae/P5912/Spain	85%	97%	ID	90%	86%	86%	88%
KX844991.1 T. gallinae/T-MA-23/Germany	92%	88%	90%	ID	96%	94%	96%
MK172846.1 T. gallinae/SAGIR-134722-A4/France	94%	84%	86%	96%	ID	95%	98%
EU881911.1 T. gallinae/P1807/Spain	94%	84%	86%	94%	95%	ID	95%
MN446018.1 T. gallinae/TG-VAN31/Turkey	95%	86%	88%	96%	98%	95%	ID

The isolates OM688823, OM688824, OM679421, OM679422, and OM688825 were 88%, 90%, 64%, 64%, and 91% identical to another Egyptian strain, LC136936.1. Moreover, the OM688823.1 isolate shared 100% identity with the German isolate, KX459442.1, and 95% identity with the Turkish isolate, MN446018.1. The OM688824.1 isolate exhibited 97% identity to the French and Spanish isolates, MK172846.1 and EU881911.1, respectively, and 96% identity to KX844991.1, isolated from Germany and MN446018.1, isolated from Turkey. The isolates OM679421.1 and OM679422.1 showed 85% identity to MT300160.1, isolated from the Kingdom of Saudi Arabia.

OM688825.1 shared 95%, 96%, 98%, and 99% identity with EU881911.1, isolated from Spain, KX844991.1, isolated from Germany, MK172846.1, isolated from France, and MN446018.1, isolated from Turkey, respectively.

The Egyptian *T. gallinae* isolates in this study appear to be similar to European (French and German) and Asian (Saudi Arabian and Turkish) isolates, probably due to some European Columbidae species (turtle dove) migrating long distances to Africa, passing through Italy, Malta, Tunisia, and through the Balkan countries, Egypt, and the Middle East. Turtle doves that breed in European Russia and Ukraine migrate mainly to Eastern Africa via Turkey and the Middle East (Marx et al. 2017).

## Data Availability

All data generated or analyzed during this study are included in this published article.
